# Short sleep recovery partially restores decision-making alterations induced by total sleep deprivation

**DOI:** 10.1093/sleepadvances/zpag038

**Published:** 2026-03-26

**Authors:** Quentin Schlitter, Vincent Beauchamps, Michael Quiquempoix, Pierre Fabries, Anthony Vacher, Marion Trousselard, Thibaut Dondaine, Fabien Sauvet

**Affiliations:** Académie militaire de la gendarmerie nationale (AMGN), Centre de recherche de la gendarmerie nationale (CRGN), 77000 Melun, France; Université Paris Cité, UMR VIFASOM, 75004 Paris, France; Université Paris Cité, UMR VIFASOM, 75004 Paris, France; REF Aéromédical departement, Institut de recherche biomédicale des armées (IRBA), 91220 Brétigny-sur-Orge, France; Académie de santé des armées (ACASAN), Ecole du Val-de-Grâce, 75004 Paris, France; Université Paris Cité, UMR VIFASOM, 75004 Paris, France; REF Aéromédical departement, Institut de recherche biomédicale des armées (IRBA), 91220 Brétigny-sur-Orge, France; Université Paris Cité, UMR VIFASOM, 75004 Paris, France; REF Aéromédical departement, Institut de recherche biomédicale des armées (IRBA), 91220 Brétigny-sur-Orge, France; Université Paris Cité, UMR VIFASOM, 75004 Paris, France; REF Aéromédical departement, Institut de recherche biomédicale des armées (IRBA), 91220 Brétigny-sur-Orge, France; REF Aéromédical departement, Institut de recherche biomédicale des armées (IRBA), 91220 Brétigny-sur-Orge, France; Académie de santé des armées (ACASAN), Ecole du Val-de-Grâce, 75004 Paris, France; École des Psychologues Praticiens, Catholic Institute of Paris, EA Religion, culture et société, 75004 Paris, France; Université de Lorraine, Inserm, INSPIIRE, F-54000 Nancy, France; University de Lille, Inserm, CHU Lille, Lille Neuroscience & Cognition, Translational Pharmacological Modulation for Neurodegenerative and Neurovascular Diseases, Department of Pharmacology, Faculty of Medicine, 59045 Lille, France; Université Paris Cité, UMR VIFASOM, 75004 Paris, France; REF Aéromédical departement, Institut de recherche biomédicale des armées (IRBA), 91220 Brétigny-sur-Orge, France; Académie de santé des armées (ACASAN), Ecole du Val-de-Grâce, 75004 Paris, France

**Keywords:** sleep deprivation, recovery, decision-making, reaction time, inhibition, decision diffusion model

## Abstract

The dynamics of recovery from impaired decision-making after total sleep deprivation (TSD) are not well understood. We investigated the impact of TSD and subsequent 3 h sleep recovery night period on decision-making. After one baseline night (8 h Time in Bed [TIB], 23:00–07:00), 40 study participants (50 per cent women) followed a sleep deprivation protocol including one night of TSD (40 h continuous awakening), followed by 3 h TIB sleep recovery period (23:00–02:00) and 8 h TIB sleep recovery period. Indices derived from reaction time (RT), Go/No-Go, and complex Go/No-Go tasks (involving perceptual components, motor responses, RT, and accuracy) were assessed daily during dual-choice decision-making tasks (MindPulse Digital Battery). Composite indices to describe executive speed, reaction to difficulty, speed/accuracy balance, and parameters from decision diffusion model analysis were recorded. Errors and RT increased after TSD and remained elevated after a 3-h sleep recovery night, particularly for Go/No-Go tasks. Anticipation and inhibition errors as well as speed/accuracy balance are not restored by 3 h sleep recovery night, whereas RT was restored. Lower decision diffusion model drift values (i.e. slower information accumulation) observed in the higher difficulty (complex Go/No-Go) after TSD, persisted after the 3-h recovery night. All parameters were restored after an 8-h TIB recovery night. No effect of sleep loss on executive speed and reaction to difficulty was observed. In conclusion, short sleep recovery partially restored decision-making alterations induced by TSD. Slower perceptual and motor processes that persist after a short recovery night may favor errors, with possible operational consequences for shift or on-call workers.

**Clinical trial:** NCT05924737, https://clinicaltrials.gov/study/NCT05924737, 2023_RECOPS study, registered 2023-06-12 (Study start 2023-10-01).

**Clinical Trial Informations:** Delta Waves and Cognitive Recovery (RECOPS), NCT05924737, https://clinicaltrials.gov/study/NCT05924737, study record: 2023-06-20, study start: 2023-10-01.

**Statement of Significance**

Sleep deprivation altered decision-making performance. In this work, we observed for the first time that a short sleep recovery (3 h Time in Bed) partially restored decision-making alterations induced by total sleep deprivation. The most complex tasks remained affected by sleep deprivation. The slowing down of perceptual and motor processes that persisted after a short night of recovery may have increased errors in decision-making, with possible operational consequences for shift workers or on-call workers.

## Introduction

Decision-making is a fundamental adaptive and cognitive process that allows an individual to choose one option among several options [1]. In high-stress environments, such as military operations, aviation, and emergency services, decision-making processes, involve complex cognitive processes and situational factors [2, 3].

External factors that influence decision-making include time pressure, uncertainty, and the complexity of information [2, 4]. Sleep deprivation is a particularly important external factor and is commonly recognized as leading to reduced attention, alertness, and responsiveness [5, 6]. However, sleep loss does not appear to affect cognitive processes globally [7] and insufficient sleep affects different components of cognitive functioning in different ways. In many situations, decisions must be made under conditions of sleep deprivation, posing significant challenges for individuals who must rely on these cognitive abilities in emergency situations [8, 9].

Overall, previous studies showed an impairment of decision-making following a sleep deprivation [5, 6, 10, 11]. Indeed, sleep deprivation reduces regional cerebral metabolism within the prefrontal cortex (PFC), the brain region most responsible for higher-order cognitive processes, including judgment and decision-making [10, 12]. In particular, sleep deprivation impaired the ability to evaluate risks and rewards, which is crucial in both simple and complex tasks [10, 13]. In their study, Killgore et al. [10] found that sleep deprivation significantly impaired decision-making in the Iowa Gambling Task, a common measure of decision-making that involves balancing short-term rewards against long-term consequences. The participants demonstrated a preference for riskier choices after 24 h without sleep.

Other approaches provide a better description of the processes underlying cognitive disorders caused by sleep deprivation or lack of sleep, such as changes in the decision diffusion model (DDM) [14]. This method of analysis, validated in numerous cognitive psychology studies [4, 15], provides a better understanding of how cognitive and emotional functions influence decision-making. Indeed, the DDM, a stochastic accumulator model, captures the complex relationship between choice and reaction times (RTs). It takes into account not only speed and accuracy, but also their interactions. A primary DDM output is the reduction in drift rate, which indicates slower and less efficient information accumulation during decision-making tasks [14–16]. Additionally, sleep deprivation can lead to increased decision boundaries, suggesting more cautious decision-making [16], and increased non-decision time, reflecting slower perceptual and motor processes [17].

To date, only a limited number of studies have examined the restoring effects of recovery sleep on cognitive and brain function that are implied in decision-making [18], even though understanding the benefits and limitations of short periods of recovery sleep is crucial for many professions that operate in emergency contexts (e.g. the military, police officers, firefighters, and first responders) [19].

Recent studies have suggested that a period of restorative sleep limited to 3  h in the middle of the night reflects typical situations encountered during prolonged operations in a military or civilian contexts [20, 21], particularly for remotely piloted aircraft pilots and fighter pilots [22, 23]. This recovery period of 3 h has been identifying as sufficient to partially mitigate altered performances, in particular sustained attention, after total sleep deprivation (TSD) [23].

In our work, we aimed to assess the recovery capacity of subjects with a short sleep period (3 h Time in Bed [TIB]) following TSD. The global number of errors to the MindPulse has been considered as the primary endpoint. Secondly, we evaluated RTs in various tasks, as well as composite indices describing executive speed (ES), reaction to difficulty (RD), balance between speed and accuracy, and DDM parameters to assess the speed of information accumulation processing during multiple-choice decision-making tasks.

## Materials and Methods

### Subjects

Forty-five healthy volunteers were recruited for this protocol. Study participants were free from medical, psychiatric, and sleep disorders. Other exclusion criteria included physical or mental health troubles based on (I) Hospital Anxiety and Depression scale (A or D score > 7), (II) significant medical history, (III) Epworth Sleepiness Scale, ESS > 10 [24, 25], (IV) Pittsburg sleep quality index, PSQI > 7 [25], (V) French version of the morningness-eveningness questionnaire, < 7 or >25, [26, 27], (VI) habitual time in bed per night <6 h. We also excluded study participants younger than 18 years and older than 45 years, with a BMI greater than 30 kg/m^2^, working at night or in shift work or jet lag (>3 time zones) in the previous month, drinking more than one glass of alcohol per day more than 5 days a week, and abusing tobacco (>10 cigarettes/day). The education level was recorded using the International Standard Classification of Education, ISCED from UNESCO, 1997 version.

All procedures were carried out in accordance with relevant guidelines and regulations, including the Declaration of Helsinki. The study received the agreement of the Est III CPP (Nancy) Ethics Committee (N° IDRCB 2023-A01221-44). It was conducted according to the principles expressed in the Declaration of Helsinki of 1975, as revised in 2001 and declared to the French National Agency for Medicines and Health Products Safety. Written informed consent was obtained from all participants, who received financial gratification for study completion. This work was a part of a protocol recoded in the Clinical Trial Database (NCT05924737).

### MindPulse test

**Figure 1:**
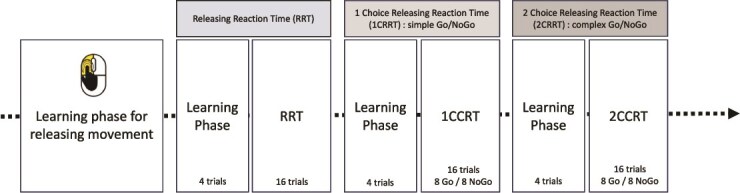
Description of test process. A first learning phase of the mouse release movement. The next phase is a “no criteria” go. Another learning phase to allow a good understanding of the instruction. Four trials will be presented and 3 must be passed to proceed to next step. This step still without criteria offers 16 trials in total. Any results we move to the next phase with a simple Go/No-Go. Like before, a phase of understanding with a 3 success on 4 trials, a phase of test with 8 Go and 8 No-Go. Finally, a complex Go/No- Go with 2 criteria conclude the test. The organization of this phase is identical as before.

In many studies, decision-making has been assessed independently of attentional processes. MindPulse test [28, 29] was developed in order to provide the ability to measure four axes of the speed-precision trade-off inherent in a subject’s fundamental decision-making: perceptual-motor speed, ES, subject accuracy, and RD [28, 29], using an increased difficulty sequence of tests (Figure 1).

The Mindpulse computed test consists of a series of increasingly difficulty decision-making tasks (It’s Brain, Bures-sur-Yvette, France, https://itsbrain.mindpulse.net/en last access on 8 May 2025) [28, 29].

Study participants were seated in front of a computer at a distance of ~60 cm from the screen and were encouraged to use their preferred hand. The test consists of four main parts (Figure 1). Before each part, a training session was conducted, in order to ensure that the instructions have been well understood. The study participants were allowed to practice as many times as necessary.

**Figure 2:**
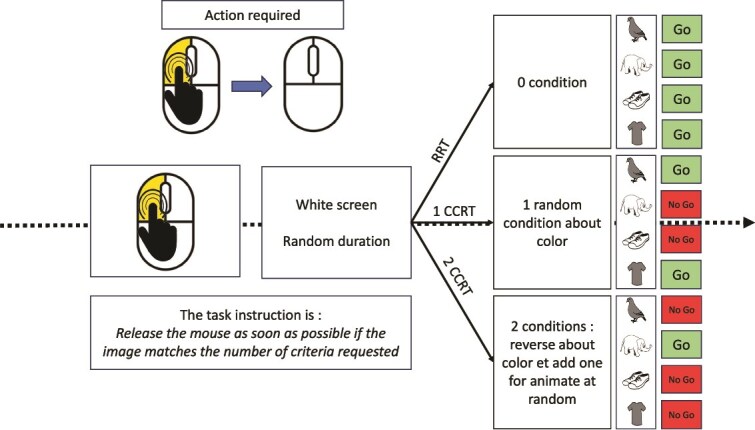
Description of the conditions. Participants were instructed to release the mouse button when the stimulus matched the given instruction. In the first phase, there are no constraints on the images, so the mouse must be released in all cases (RRT, simple reaction time). Then there is a condition on the color (gray or white) which is randomized (1 CRRT, Go/No-Go task). Finally, there are two conditions to be taken into account: Inverting the color condition and randomly introducing the condition relating to the living nature of the object (2 CRRT, complex Go/No-Go task).

In each part, participants were required to respond to an image presented on the screen:

- **Preliminary, the learning the “releasing movement.**” The first task involved learning the gesture of clicking the mouse button at the signal and releasing it when an image appeared. A “clicking hand” picture prompted the participant to click on the computer mouse (see Figure 2). The experimenter explained the instructions to the participant: “Keep your finger pressed as long as the screen remains blank. Then release the mouse button as quickly as possible when an image appears.”
- **The Releasing Reaction Time (RRT) Task** measures of the participant’s RRT. Participant must disengage the motor gesture when the target image appears. This task included a learning part (4 trials and 3/4 had to be passed to proceed to the test part), followed by a test part with 16 trials (see Figure 2).
- **The 1-Choice Releasing Reaction Time (1CRRT)** is a simple Go/No-Go Releasing Task. 1CRRT provides a measurement of participant’s RT with one choice, and errors in the form of a Go/No-Go task. The category is a color choice (the study participant must release the mouse button if the image is “gray” or “white”). The target category was randomized among the study participants. Participants were instructed to release the mouse button (Go response) as quickly as possible when the picture was of the target color. They had 3 s to perform a Go response after which any response was considered a no-go response. This task included a learning part (4 trials), then a test part with 16 trials, 8 Go and 8 No-Go (Figure 2).
- **The 2-Choice Releasing Reaction Time (2CRRT)** is a Complex Go/No-Go Releasing Task. In the 2CRRT task, participant had to react (release) only for stimuli corresponding simultaneously to the two required categories. The color criteria was systematically reversed with respect to the 1CRRT, which requires inhibiting the previously relevant color. The second new criteria corresponds to the animate/inanimate nature of the picture stimuli. The selection of the relevant criterion for this new category was random. The 2CRRT included a learning part (4 trials), while the 2CRRT test part comprised 16 trials, 8 Go and 8 No-Go (Figure 2).

In each task, the participant received instructions on the screen, then performed the trials. In each trial, an image instructing the participant to press the mouse appeared, then as soon as the mouse button had been pressed, the screen turned blank for a variable amount of time (between 2 and 7 s called “waiting time”) during which the participant had to maintain the pressure on the mouse.

Each of the three tasks were preceded by a learning period with four trials. If the participant made an error, the experimenter intervened to explain it. Participants who failed more than once out of the four training trials were asked to repeat the four training trials once or twice. Then, each participant accomplished 3 × 16 actions per task, with a total completion time of around 15 min.

**Table 1:** MindPulse Output Parameters

**MindPulse task**	**Output**
RRT (8)	Reaction time (RT, ms) RT standard deviation (RTsd, ms) Anticipation errors (*n*) Omission errors (*n*) Correct response (*n*) Total number of errors (*n*) Total number of correct response (*n*) Efficiency
1CRRT (9)	Reaction Time (RT, ms) RT standard deviation (RTsd, ms) Anticipation errors (*n*) Omission errors (*n*) Choice errors (*n*) Correct responses (*n*) Total number of errors (*n*) Total number of correct response (*n*) Efficiency Drift rate (DDM_1C_drift) Boundary (DDM_1C_B)
2CRRT (11)	Reaction time (RT, ms) RT standard deviation (RTsd, ms) Anticipation errors (*n*) Omission errors (*n*) Choice errors (*n*) Inhibition errors (*n*) Overload errors (*n*) Combined errors (*n*) Total number of errors (*n*) Total number of correct responses (*n*) Efficiency Drift rate (DDM_2C_drift) Boundary (DDM_2C_B)
Global (6)	Mean RT (ms) Mean RT standard deviation (RTsd, ms) Global number of errors Global number of correct responses Executive speed (ES) Reaction to difficulty (RD)

The test measures the releasing RT equal to the latency in releasing the mouse button after the image appears. The accuracy and types of errors collected by MindPulse are shown in Table 1:

- **Anticipation error**: participant releases pressure on the mouse button before the image appears or within 100 ms after the image appears on the screen.
- **Omission error:** participant did not release within 3 s after the image matching the criteria and kept pressing the button.
- **Choice error:** In the two Go/No-Go Tasks, a choice error is when the participant is releasing the pressing button on a wrong stimulus that doesn’t fit the criterion. Choice errors are active errors where the participant actively responds to a wrong image. Within the choice errors category, three subcategories were created depending on which criterion was not met by the stimulus. Those subchoices errors are specific to the 2CRRT including: **Inhibition error** (e.g. the participant releases the pressing button on an image that is not the right color), **Overload error** (e.g. the participant realizes the pressing button on a picture that doesn’t meet the alive/not alive criterion) and **combined error** (e.g. both on the color and on the alive/non-alive criteria).
- **Total of errors,** that is the sums of all the errors made in the three parts of the task, whatever their types or subtypes, was calculated. The total of error is the primary endpoint.

Moreover, we calculated for each level the RT variation index (standard deviation), which is very sensitive to extreme values and its reliability is low for individual RT measurements [30]. Nevertheless, it is an interesting measure which reflects fluctuations in attentional and executive control as well as impairments in information processing and, in particular, a dysfunction related with a failure to maintain attentional control [31].

RT can be divided into different components that are not all affected by the same factors. Traditionally, RT is described as having a perceptual and psychomotor component, as well as a more complex “executive” component, measured with an “executive speed” usually described as the more advanced cognitive, executive processes involved in a decisional task, such as Go/NoGo. Psychomotor speed is more related to the time needed to perceive and process a basic motor response to a stimulus, such as simple RT tests. Motor planning itself consumes a very small fraction of the RT. RD corresponds to the subject's adjustment when faced with difficulty. RD provides insight into how the study participant adapts to perceived difficulty (e.g. in relation to their emotional state when faced with the situation) with a numerical measurement of the impact of this adaptation/maladaptation on their executive cognitive speed. Therefore, the differentiation of each component of RT is necessary to better understand the mechanisms underlying slowing and to avoid false positives or false negatives [28, 29]. Moreover, the efficiency score (ES), a metric that incorporate speed and accuracy (correct responses) may help to assess whether accuracy is being sacrificed for speed (or vice versa) [32]. Changes in accuracy (speed) can be offset by changes in speed (accuracy) to avoid or neutralize trade-offs between speed and accuracy.

The MindPulse software can therefore automatically calculate the following parameters (for a full description, refer to Suarez et al. [28]):

- ***Simple reaction time (SRT)*** index of a participant is its mean age- and sex-corrected RT of the RRT Task. The SRT is mostly linked to perceptual-motor time and serves as a baseline for other indices.
- ***ES*** (1) is the average executive time after subtracting the SRT and using the difficulty rescaling 1.68 for the Complex Go/No-Go 2 Choice task (2CRRT) [28]. The formula to define the average executive time is:

ES = average of (1CRRT_RT − SRT) + (2CRRT_RT − SRT)/1.68

- ***RD*** defined (2) as the difference between the rescaled Complex Go/No-Go-SRT (2CRRT) and the Simple Go/No-Go-SRT (1CRRT). However, this difference is not a homoscedastic variable, meaning its variance is proportional to the executive time itself. To create a homoscedastic variable, the ratio RD is defined as:

RD = (2CRRT_RT − SRT)/1.68 − (1CRRT_RT − SRT))/(2 × ES)

- ***ES*** (Reaction time/correct response) has been also calculated [32].

At the end, MindPulse provides also scores based on the parameters of the DDM, which integrates RT and accuracy data into a single set of performance indicators. Two DDM parameters were calculated:

- ***Drift rate*** that represents how efficiently an individual processes information to make a correct decision. Drift rate has been calculated for the 1CRRT (DDM_1C_drift) and 2CRRT (DDM_2C_drift).
- ***Boundary (B) separation*** (denoted DDM_1C_B for 1CRRT and DDM_2C_B for 2CRRT) was calculated. This parameter reflects an individual's speed-accuracy trade-off settings, which can be inherent or influenced by strategic choices. Higher boundary values prioritize accuracy over speed, leading to a more cautious and slower decision-making process. Conversely, lower values prioritize speed over accuracy. A larger boundary separation requires more evidence before a decision is made, resulting in higher accuracy but slower responses.

### Study design and testing conditions

Before the experiment, participants were asked to complete a sleep/wake diary and maintain a regular habitual sleep during the 10 days before the experiment. Sleep/wake patterns were checked using wrist actigraphy (Actiwatch TM, Cambridge Neurotechnology, Cambridgeshire, UK). They were also asked to have a caffeine consumption of less than 200 mg during this phase.

**Figure 3:**
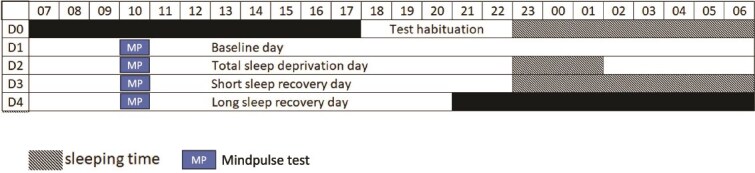
Protocol in the laboratory. MindPulse tests were made every day at 10:00. BS is the baseline night (8 h TIB), R1 is the restricted recovery night (3 h TIB), and R2 is the long recovery night (8 h TIB). Black periods indicate the time before and after study onset.

During the protocol (Figure 3), participants were housed 4 days in the laboratory including a night with an 8 h TIB (23:00 to 07:00), a sleep deprivation protocol including one night of TSD (i.e. 40 h of continuous awakening), following by a 3-h TIB sleep recovery period (23:00 to 02:00), then another 8 h TIB recovery night (23:00 to 07:00).

This study was conducted at the Armed Forces Biomedical Research Institute (IRBA) in Brétigny-sur-Orge, France. The ambient temperature was maintained at 22 ± 1°C. Light intensity was between 150 and 200 lux during waking periods and all lights were turned off during sleep periods. In the laboratory, participants were not allowed to engage in physical exercise or to consume coffee, alcohol, or other psychoactive substances during the study, in order to prevent sleep disturbances. They were under visual surveillance of research staff members. In addition, they wore a wrist actigraphy to check that they stayed awake during the sleep restriction. When they were not engaged in testing, meals, or sleep periods, participants were allowed to read, to watch videos, or to speak with other participants or staff members and play board games, following a pre-established program. Smokers were allowed to smoke one cigarette immediately after each meal (a maximum of three cigarettes per day) in order to limit the effects of possible withdrawal symptoms. Participants’ performances were assessed with MindPulse at 10:00 every day.

### Statistical analyses

Statistical analyses were computed using Jamovi (Version 2.3.28, The Jamovi project (2025), retrieved from https://www.jamovi.org, lasted access on May 3, 2025). Values were expressed as mean ± 95% confidence interval (95% CI). For MindPulse outputs, we lead a two factors linear mixed model with the day (ordinal four levels factor) and difficulty (ordinal three levels factor). Participants were added as a random factor in order to create a repeated measure model. Effect sizes were estimated with calculation of the eta square (η^2^ > 0.01 indicates a small effect, η^2^ > 0.06 a medium effect and η^2^ > 0.14 a large effect). In the case of significant main effect, differences between days were tested using Bonferroni post-hoc tests, in order to manage multiple comparisons biases. Type of error values were compared using a linear mixed model with day as an ordinal factor (four levels) and participant as a random factor.

In order to identify possible influence of covariables on RT or global number of errors, we tested effect of sex and laterality. If a significant effect was observed, we assessed interactions with day and difficulty with new linear mixed models included the covariable.

Correlations were made using the calculus of the Pearson *r* coefficient. Corrected *p* value <.05 was considered statistically significant.

## Results

Of the 45 initial participants, two withdrew their consent and did not participate in the protocol, two were excluded from the analyses for medical reasons discovered during the study, and one participant was excluded due to performance significantly lower than the other participants (number of errors >2 standard deviations from the first day).

Finally, 40 healthy participants (20 women and 20 men, 4 left-handed, age: 32.3 ± 2.3 years old, weight: 73.4 ± 4.0 kg, height: 171.2 ± 3.2 cm) were considered for the analysis. The average sleep time in the seven days preceding the experimental phase was 478.5 ± 15.2 min. During the laboratory protocol, the averaged TST were 469.2 ± 4.1 min during the D1 to D2 night, 179.9 ± 2.1 min during the D2 to D3 night, and 477.0 ± 2.1 min during the D2 to D3 night. Seven participants had an upper secondary degree, 11 a post-secondary non-tertiary degree, 7 a short-cycle tertiary degree and 15 a bachelor or equivalent diploma.

### Reaction times

**Figure 4:**
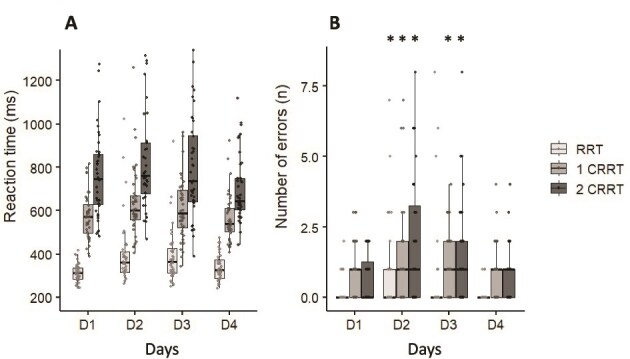
(A) Reaction time. (b) Number of error taking account the difficulty and days. ^*^Difference with D1 (*p* < .05) for the same difficulty level.

We observed a significant difficulty effect (*F*_(3, 123)_ = 4.12, *p* = .008, η^2^ = 0.16), characterized by an increase in RT of 208 ± 80 ms as task difficulty increased (Figure 4A; *p* = .02 between 2CRRT and RRT). No significant difference was observed between RRT and 1CRRT. Moreover, the increase in RT with increasing difficulty has been observed each day of testing.

Without interaction, we also observed a significant day effect for the global Mean RT RT (*F*_(3, 123)_ = 9.54, *p* < .001, η^2^ = 0.18), with higher values during D2 (*p* = .001) and D3 (*p* = .001) in comparison to D1. During D4, values were lower than in D3 (*p* = .03). No difference was observed between D2 and D3 (*p* = .54) and between D1 and D4 (*p* = .25).

We observed a significant day effect on the global RT standard deviation (*F*_(3, 123)_ = 5.98, *p* = .001, η^2^ = 0.16) without difficulty effect (*F*_(3, 123)_ = 2.04, *p* = .11, η^2^ = 0.05). RT standard deviation increased in D2 (*p* = .003) and D3 (*p* = .03) in comparison to D1. No difference was observed between D1 and D4 (*p* = .78) and between D2 and D3 (*p* = .54).

No correlation was observed between RT and education level (*r* = 0.09, *p* = .82) or age (*r* = 0.09, *p* = .23). We didn’t observe laterality (*p* = .67) or sex (*p* = .75) effects.

### Global number of errors

We observed a significant day main effect for on the global number of errors (*F*_(3, 123)_ = 10.09, *p* = .001, η^2^ = 0.18), with higher values during D2 (*p* = .001) and D3 (*p* = .047) in comparison to D1. No difference between D2 and D3 (*p* = .32) and between D1 and D4 (*p* = .98) were shown in Figure 4.

We also observed a main difficulty effect (*F*_(2, 123)_ = 5.21, *p* = .02, η^2^ = 0.14) and an interaction between difficulty and day (*F*_(6, 123)_ = 3.92, *p* = .023, η^2^ = 0.16). In RRT conditions, we observed an increase of the number of errors during D2 in comparison to D1 (*p* = .03) and D3 (*p* = .04) (Figure 4B). No difference was observed between D1 and D4 (*p* = .32). In the 1CRRT and 2CRRT conditions, we showed an increase of the number of errors during D2 (*p* = .04 and *p* = .03, respectively) and D3 (*p* = .04 and *p* = .03, respectively) in comparison to D1. No difference was observed between D1 and D4 (*p* = .26).

No correlation was observed between the number of error and education level (*r* = 0.01, *p* = .92; *r* = 0.09, *p* = .82) or age (*r* = 0.09, *p* = .23). We didn’t observe laterality (*p* = .78) or sex (*p* = .43) effects.

For the global ES and the RD, we didn’t observe a significant day effect (*F*_(3, 123)_ = 1.59, *p* = .22, η^2^ = 0.04 and F_(3, 123)_ = 0.22, *p* = .87, η^2^ = 0.01, respectively).

### Types of errors

#### R‌RT task

**Figure 5:**
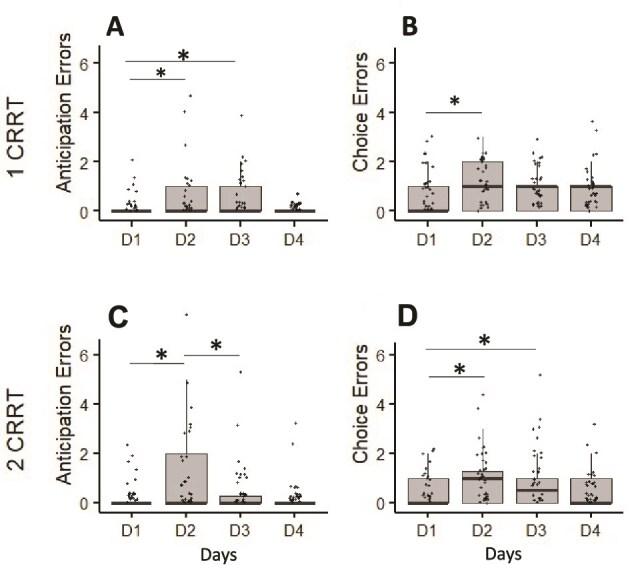
Type of errors (anticipation and choice) for the 1CRRT and 2CRRT tasks. ^*^Difference with D1, *p* < .05. $Difference between D3 and D2, *p* < .05.

For Anticipation errors, we observed a day main effect (*F*_(3,123)_ = 3.84, *p* = .01, η^2^ = 0.08), with a significant increase during D2 compare to D1 (*p* = .03). During D3, values returned to baseline, with no difference with D1 (*p* = .91) and D4 (*p* = .34). No day effect has been observed for omission error (*F*_(3,123)_ = 1.01, *p* = .64) (Figure 5).

#### 1CRRT task

A day effect has been observed for anticipation errors (*F*_(3, 123)_ = 4.09, *p* = .008, η^2^ = 0.16). Values in D2 (*p* = .01) and D3 (*p* = .04) were higher than D1 (Figure 5). No difference was observed between D1 and D4 (*p* = .62) and between D2 and D3 (*p* = .45). No day effect has been observed for choice errors (*F*_(3, 123)_ = 1.24, *p* = .34), nor for and omission errors across days (*F*_(3, 123)_ = 1.21, *p* = .25).

#### 2CRRT task

For anticipation errors, we observed a day effect (*F*_(3,123)_ = 6.87, *p* = .001, η^2^ = 0.20), with a significant increase during D2 (*p* = .01) compared to D1. Values decreased in D3 compared to D2 (*p* = .005). No difference was observed between D1 and D4 (*p* = .55) and between D3 and D4 (*p* = .30).

For choice errors, we observed a day effect (F_(3,123)_ = 4.21, *p* = .006, η^2^ = 0.14), with a significant increase during D2 (*p* = .01) and D3 (*p* = .01) compared to D1. No difference was observed between D1 and D4 (*p* = .55) and between D2 and D3 (*p* = .35).

Considering omission errors, no day effect has been observed (F_(3, 123)_ = 1.12, *p* = .44). The number of overload and combined errors was very low (0.516 ± 0.810 and 0.0755 ± 0.265) and no day effect has been observed.

### Efficiency scores

**Figure 6:**
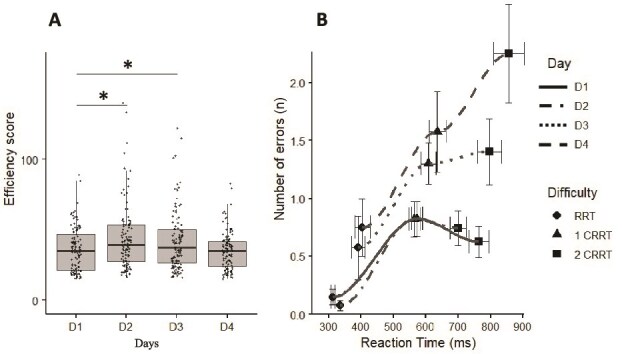
(A) Efficiency during the days and (B) speed accuracy/balance. ^*^Difference versus D1, *p* < .05.

Considering ES, we observed an interaction between days and difficulty (*F*_(6, 123)_ = 3.98, *p* = .012, η^2^ = 0.18) with higher values during D2 (*p* = .02) and D3 (*p* = .04) compared to D1 (Figure 6A). We showed (Figure 6B) that the speed-accuracy balance increased during D2 and D4, in particular during the 2CCRT task.

### D‌DM parameters

**Figure 7:**
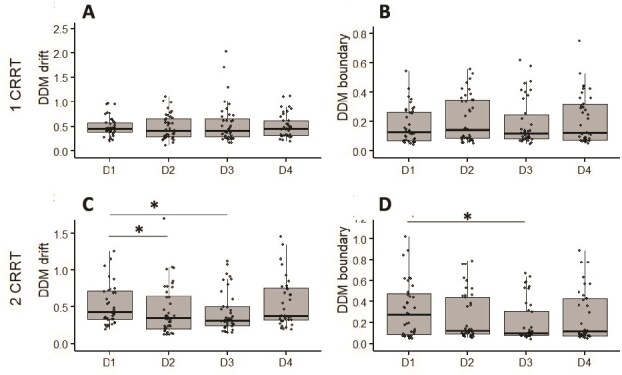
Decision diffusion model parameters (drift and boundary, for 1CRRT (A: DDM_1C_drift and B: DDM_1C_B) and 2CRRT (C: DDM_1C_drift and D: DDM_1C_B). ^*^Difference versus D1, *p* < .05.

For 1CRRT (Figure 7A and B), we observed no significant day effect on the DDM_1C_drift (*F*_(3, 38)_ = 0.47, *p* = .7) and DDM_1C_B (*F*_(3, 38)_ = 0.24, *p* = .86) indices. For the 2CRRT task (Figure 7C and D), DDM_2C_drift decreased on D2 (*p* = .03) and D3 (*p* = .02) (day effect, *F*_(3, 38)_ = 3.04, *p* = .03, η^2^ = 0.14), whereas DDM_2C_B was lowered only on D3 compared to D1 (*p* = .03) (day effect, *F*_(3, 38)_ = 2.20, *p* = .04, η^2^ = 0.10).

### Correlations between parameters

We didn’t observe a sex effect on global number of errors (*p* = .21) or RT (*p* = .12). Moreover, no correlation has been found between age and analyzed parameters. The number of errors was correlated with the reaction time (*r* = 0.62, *p* = .0001) and the DDM drift parameters (*r* = 0.25, *p* = .01 for 1CCRT and *r* = 0.31, *p* = .01 for 2CCRT). The DDM drifts were also correlated with the number of anticipations errors (*r* = 0.21, *p* = .01 for 1CCRT and *r* = 0.25, *p* = .01 for 2CCRT), whereas no correlation was observed with boundary parameters.

## Discussion

The aim of our study was to analyze the recovery dynamics of decision-making after TSD, followed by a short recovery period of 3 h. Our results showed that a short recovery after sleep deprivation is not sufficient to fully restore complex decision-making abilities (reflected by a decrease of performance), whereas vigilance and selective attention appear to recover more rapidly.

To study the beneficial impact and limitation of a short recovery night, we chose a restricted 3 h TIB duration followed by a long 8 h recovery night. This design may reflect situations encountered during sustained operations, as it has been previously published in a military environment including remotely piloted aircraft and combat pilots [22, 23]. These authors observed that restricted recovery partially mitigate altered performances that are fully restored after an 8-h night's sleep. In particular, sustained attention seems to be restored by 3 h restricted sleep, whereas executive processes are not [23]. Many other professionals (firemen, healthcare professionals, policemen, etc.) have to deal with these situations of reduced sleep opportunities periods after sleep deprivation [33], with consequences on health and performance. A restricted recovery night of 3 h still allows for nearly two complete sleep cycles, characterized by a high proportion of deep sleep, which is considered restorative due to the decrease of homeostatic pressure [34, 35].

Several studies have evaluated the effects of sleep debt on various levels of attention and decision-making using different tests [6, 10, 36]. In our study, we provided a better understanding of the effects of sleep debt and recovery by using a task that evaluates decision-making at increasing levels of difficulty within the same paradigm (MindPulse). This provided a better understanding of the effects of sleep debt and recovery on decision-making. Some parameters calculated from MindPulse were not affected by TSD. In particular, the response to difficulty and ES, which are affected by various clinical pathologies. In this study, performance remained within the expected norms for healthy individuals of their age [29].

Some studies indicated that partial sleep deprivation does not consistently impair decision-making abilities, although it can lead to increased impulsivity and risk-taking behaviors in certain situations [37]. Studies have shown that minor reductions in sleep, such as losing 1 h of sleep per night or sleeping only 5–6 h per night over several nights, do not significantly affect the performance on standard decision-making tasks in healthy participants [38].

However, evidence suggests that partial sleep deprivation can cause cautious people who prefer to gather extensive information before making decisions to act impulsively and take risk [11]. This effect was less pronounced than during TSD [36]. Additionally, partial sleep deprivation may reduce behavioral inhibition, which is the ability to suppress inappropriate responses, but it does not seem to impact more complex decision-making processes such as delay discounting or risk preference [39]. So, our results confirm that a single night of TSD (24 h of continuous awakening) can impair decision-making in healthy participants, particularly when the task becomes complex, in association with a risky comportment as evidenced particularly by the DDM parameters [5, 6, 11, 13, 28, 36, 40]. Many other studies observed these results during more intense sleep debt [10, 12].

In our work, we have highlighted that partial sleep recovery, which involves regaining only some of the lost sleep after a period of deprivation, had various effects on decision-making in healthy participants. In a recent study, using the same duration of recovery nights protocoled after TSD, it has been observed that while 3 h of sleep have improved emotion regulation and latency to response inhibition, attention performance required 8 h of sleep to alleviate the observed 70 per cent impairment [23]. However, after TSD involving staying awake for more than 36 h, we showed that at least 9 h of recovery sleep is needed in order to recover a level of performance close to the baseline level for a sustained attention task [41–44] or a task assessing inhibitory capacity [45], which generally represents a longer sleep time than the participants' usual sleep time.

Moreover, we observed that anticipation and choice errors were the main type or errors observed after sleep deprivation, whereas omission errors are not increased [46]. These results could be linked to an increase impulsivity triggered by impaired functional connectivity between the PFC and the cerebral cortex, which in turn predicts the risk-taking behavior found after sleep deprivation [47]. In our study, we observed after the short recovery sleep, persistent errors, particularly for complex cognitive tasks, when participants had to perform two categorizations (2CRRT), despite the correction of attentional performance and an increase in reaction time that was insufficient to compensate for the loss of efficiency. This result confirms that executive functions recover more slowly than attentional functions as sustained attention or vigilance. This is an important conclusion that justifies the assessment of complex executive functions to study recovery kinetics in order to determine adequate rest times. Furthermore, similar studies with different recovery sleep durations would allow for more accurate determination of the recovery times required for each level of difficulty of complex tasks.

Our results are consistent with those reported in the literature, showing a decrease in the drift rate only for the most executive task (i.e. 2CRRT in our work) after sleep deprivation [14, 15]. Nevertheless, we show for the first time that these values remained low despite 3 h of recovery sleep. This decrease in drift values suggests a decrease in the quality or speed of information processing in the executive task [48]. The drift rate appears to be the most sensitive parameter to sleep deprivation and recovery. Lower drift rates are predictive of a greater vulnerability to performance decline following sleep loss [14, 17, 49]. Recovery sleep has the potential to restore the drift rate, although the speed and completeness of this recovery vary depending on whether the sleep loss was acute, such as total deprivation, or chronic, such as restriction [14, 49, 50]. These results may be linked to impulsivity and the decline in the quality of information processing induced by sleep deprivation, and that remains altered after a restricted recovery night.

Decision boundaries, which relate to response caution, are less consistently affected, but there is some evidence indicating they may widen during deprivation and return to normal with recovery. Our results are in line with previous studies showing a decrease in boundary [17]. The decrease in boundary for the most effortful task, observed after the restricted recovery night, reinforces the hypothesis of impulsivity.

However, our work has many limitations. First, we did not have a control group, and we cannot rule out possible bias caused by repeating the tests day after day. Nevertheless, the absence of any difference between the first and last days is reassuring. Moreover, our observations must be considered in the context of the overall time taken for the test and the number of trials. There is no standard number of trials for Go/NoGo tests. In our protocol, with 144 trials, we are in line with publications that highlight the effects of sleep deprivation on Go/NoGo tasks (i.e. between 60 and 100 trials, with an 80/20 ratio) [51]. Nevertheless, it is plausible that the effects of degradation are only felt after a certain period. Comparing these results with other findings from the experiment, we can see that degradation evolves over time [52]. Thus, it is reasonable to assume that more complex tasks will be affected, but only after a certain duration. In our work, we did not perform any electrophysiological measurements. Future studies could assess the impact of restricted recovery on alterations in the functional connectivity of the PFC in order to evaluate their involvement in increased impulsivity.

In a precedent work with a similar design, the adverse effect of sleep deprivation was different across the units, and the attention performance of both the helicopter pilots and the remotely piloted aircraft was significantly more affected compared to combat pilots following 3 h of sleep in 2 days [23]. Moreover, given a certain amount of sleep time, there appears to be significant inter-individual variability in the ability to restore inhibitory capacity [45]. Future studies will need to be conducted to assess the relevance of our results in different populations and in more ecological contexts. Moreover, conducting several assessments throughout the day would also have made it possible to evaluate the impact of the circadian rhythm and measure the duration of the restorative benefits of 3 h of nighttime sleep.

## Conclusion

Our results are a meaningful step toward understanding how decision-making processes are partially restored following short sleep opportunities after TSD. Indeed, a 3-h recovery sleep night is not sufficient to restore decision-making alteration induced by TSD, despite its beneficial effects on attentional performance. Slower perceptual and motor processes that persist after a short recovery night may favor errors, with possible operational consequences for shift or on-call workers (firemen, soldiers, pilots, etc.). It is essential to recommend longer recovery times whenever possible or to suggest strategies for optimizing recovery. MindPulse test has emerged as a useful tool for assessing the effects of recovery on different levels of decision-making and risk-taking behavior. This tool could offer prospects for evaluating sleep debt optimization strategies, such as extending sleep prior to debt, naps, or cognitive training, and identifying participants who require longer recovery times.

## Data Availability

Data are available by request to the corresponding author (V.B.).
